# Comparison of 24-2 and 24-2C Visual Field Testing in Eyes with Ocular Hypertension

**DOI:** 10.3390/medicina62101962

**Published:** 2026-10-11

**Authors:** Berire Şeyma Durmuş Ece, Kaan Özkan, Halime Kübra Şahin, Musab Özdere

**Affiliations:** Department of Ophthalmology, Faculty of Medicine, Kastamonu University, Kastamonu 37150, Turkey; kozkan@kastamonu.edu.tr (K.Ö.); hksahin@kastamonu.edu.tr (H.K.Ş.); muhammed.ozdere@saglik.gov.tr (M.Ö.)

**Keywords:** ocular hypertension, 24-2C, standard automated perimetry, central visual field defects, glaucoma

## Abstract

*Background and Objectives*: Ocular hypertension (OHT) is associated with a variable risk of progression to primary open-angle glaucoma, and early detection of functional abnormalities is important during follow-up. The 24-2C visual field pattern was designed to improve central sampling by adding 10 test locations to the conventional 24-2 grid. This study aimed to compare central visual field findings obtained with the 24-2 and 24-2C protocols in patients with OHT. *Materials and Methods*: This prospective, single-center, paired comparative study included 60 eyes of 60 patients with OHT. Each eye underwent 24-2 and 24-2C testing using the SITA Faster strategy during the same visit, with randomized test order. Global indices, test duration, the number of abnormal points within the central 10°, central mean sensitivity, and the presence of abnormal central clusters were compared between protocols. Peripapillary retinal nerve fiber layer (RNFL) and macular ganglion cell complex (mGCC) thicknesses were measured using spectral-domain optical coherence tomography, and structure–function relationships were explored. *Results*: Mean deviation, pattern standard deviation, visual field index, false-positive rate, and central mean sensitivity did not differ significantly between protocols. Test duration was longer with 24-2C than with 24-2 (167.4 ± 39.2 vs. 141.6 ± 31.9 s; *p* < 0.001). The number of abnormal points at *p* < 0.05 within the central 10° was higher with 24-2C (3.81 ± 3.6 vs. 2.35 ± 2.4; *p* = 0.006). Abnormal central clusters were detected in 19 eyes (31.7%) with 24-2C and 9 eyes (15.0%) with 24-2 (*p* = 0.031). Among eyes classified as normal on 24-2, 14 of 51 (27.5%) showed a central cluster on 24-2C. No significant correlations were found between central functional parameters and average RNFL or mGCC thicknesses. *Conclusions*: 24-2C detected more central abnormalities than 24-2 in eyes with OHT, while global visual field indices remained similar. Longitudinal studies are needed to establish the reproducibility and prognostic relevance of these additional findings.

## 1. Introduction

Ocular hypertension (OHT) is a clinical condition characterized by persistently elevated intraocular pressure in the absence of glaucomatous optic nerve head damage or definite visual field loss on standard automated perimetry, with a variable risk of progression to primary open-angle glaucoma (POAG) [1]. A major challenge in the management of OHT is to detect functional loss at the earliest possible stage in individuals at higher risk of conversion, while avoiding unnecessary treatment in those at lower risk. Accordingly, the ability of functional tests to detect early functional changes is important during follow-up, both for risk stratification and for guiding the timing of treatment.

Although the macula accounts for only a small proportion of the retinal area, it is of particular clinical importance because it contains approximately half of all retinal ganglion cells (RGCs) and has a disproportionately large representation in the visual cortex [2]. In the conventional 24-2 visual field pattern, test locations are evenly spaced at 6° intervals, resulting in relatively sparse sampling of the macular region. Within the central 10°, only 12 test points are evaluated, consisting of four innermost central points and eight surrounding paracentral points. In contrast, the 10-2 test pattern tests 68 locations spaced 2° apart within the central 10° [3]. Structural and functional evidence indicates that the macula and central visual field may be affected from the early stages of glaucomatous damage [4,5]. However, because the widely used 24-2 grid samples the central 10° relatively sparsely, central defects may be detected with 10-2 testing in a substantial proportion of eyes previously considered “normal” on 24-2. This finding has been reported not only in early glaucoma but also in glaucoma suspects and individuals with OHT [6]. These considerations suggest that, in a population such as OHT, in which conventional perimetric loss is generally considered to be absent, denser sampling of central visual function may be important for both follow-up and classification. Furthermore, central visual field damage is particularly relevant to vision-related quality of life and daily activities, highlighting the potential clinical importance of identifying such abnormalities at an early stage [7]. On the other hand, the additional time and workload associated with routine 10-2 testing have increased interest in more “hybrid” approaches [8]. The 24-2C grid was developed in this context by adding 10 central test locations, selected from points most frequently identified as abnormal on 10-2 testing, to the standard 24-2 pattern, with the aim of facilitating the detection of potential central defects [9]. Clinical studies have suggested that 24-2C may improve the detection of central clusters of functional loss and enhance central structure–function agreement while largely preserving global visual field indices [10]. However, it remains unclear how much additional information 24-2C provides in the central field compared with 24-2 specifically in a pure OHT population, how clinically meaningful the central abnormalities detected by 24-2C are in eyes classified as “normal” on 24-2, and to what extent these findings correspond with structural measures such as RNFL and mGCC.

This study aimed to compare central visual field findings obtained with the Humphrey 24-2 and 24-2C protocols in patients with OHT and to assess their relationship with peripapillary RNFL and mGCC thicknesses.

## 2. Materials and Methods

This prospective, single-center, paired comparative study included patients with ocular hypertension (OHT) who were under follow-up at the glaucoma clinic and were consecutively enrolled between February 2026 and April 2026. Patients presenting during the study period were screened for eligibility according to the predefined inclusion and exclusion criteria. The study was conducted in accordance with the tenets of the Declaration of Helsinki and was approved by the local ethics committee (Decision No. 2026-09; 15 January 2026). Written informed consent was obtained from all participants.

For the purposes of this study, OHT was defined as an intraocular pressure (IOP) greater than 21 mmHg, measured by Goldmann applanation tonometry on two separate visits in the study eye, together with an open anterior chamber angle on gonioscopy, no clinical evidence of glaucomatous optic neuropathy on dilated optic disc examination, and no reproducible glaucomatous visual field defect on standard automated perimetry at the baseline evaluation, independent of the comparative tests performed at the study visit. The 24-2 and 24-2C results obtained at the study visit were not used to establish the diagnosis of OHT or determine eligibility for inclusion and were evaluated solely as study outcome measures. Structural measurements obtained by optical coherence tomography (OCT), including retinal nerve fiber layer (RNFL) and macular ganglion cell complex (mGCC) parameters, were likewise not used for diagnosis or eligibility but were recorded for structural assessment and structure–function analysis.

The inclusion criteria were age ≥ 18 years, consent for the use of clinical data for research and educational purposes, absence of ocular (e.g., age-related macular degeneration or diabetic macular edema), systemic, or neurological comorbidities that could affect visual field results, and sufficient cooperation to complete perimetric testing. Eyes with high refractive errors, defined as an absolute spherical equivalent greater than 6.0 diopters or astigmatism greater than 3.0 diopters, were excluded. Additional exclusion criteria included media opacities that could compromise OCT or perimetric test quality, including clinically significant cataract, marked corneal opacity, or vitreous haze; amblyopia, strabismus, or nystagmus that could interfere with fixation; and a history of neurological disease, such as previous stroke or intracranial mass lesions. With respect to ocular surgery, patients with a history of ocular surgery other than uncomplicated cataract surgery with intraocular lens implantation, those who had undergone any ocular surgery within the preceding 6 months, and those with a history of ocular trauma were excluded.

Visual field testing was performed using the Humphrey Field Analyzer III (Model 830; Carl Zeiss Meditec, Dublin, CA, USA) with a Goldmann size III white stimulus against the standard bowl background illumination of 31.5 apostilbs. All participants were under follow-up at our clinic and had previous experience with automated perimetry. One eye per participant was randomly selected for analysis. In patients with both eyes eligible, the study eye was determined using a computer-generated right/left randomization list prepared before study initiation. Each eye underwent two visual field protocols during the same visit: standard 24-2 and 24-2C, both performed with the SITA Faster strategy. To minimize fatigue and learning effects, test order was randomized at the patient level, with half of the participants undergoing 24-2 first and the other half undergoing 24-2C first. A rest period of 10–15 min was provided between tests. Tests with a false-positive rate ≥ 15% or technician-identified artifacts were excluded from the analysis.

For each test, mean deviation (MD, dB), pattern standard deviation (PSD, dB), visual field index (VFI, %), Glaucoma Hemifield Test (GHT) classification, test duration, and the number of abnormal test points within the central 10° were recorded. Mean sensitivity across all test locations within the central 10° was also calculated for both test patterns. Because sensitivity values expressed in decibels are logarithmic, pointwise sensitivity values were first converted to a linear scale (10^dB/10^). The linear sensitivity values were then averaged, and the resulting mean was converted back to decibels using 10 × log_10_ (mean). Central visual field results were also classified as normal or abnormal according to a predefined cluster criterion. For the 24-2 protocol, the 12 test locations within the central 10° were evaluated, whereas for the 24-2C protocol, a total of 22 central locations were assessed, comprising the same 12 locations plus the 10 additional central points unique to 24-2C. A central visual field was considered abnormal according to the cluster criterion when at least three adjacent points within the central 10° were abnormal at *p* < 0.05, with at least one of these points abnormal at *p* < 0.01. Eyes not meeting this criterion were classified as having a normal central visual field according to the cluster criterion. Because of the asymmetric distribution of test locations in the 24-2C pattern, adjacency was defined based on the spatial arrangement of test points on the visual field grid. Test locations were considered adjacent when they were directly neighboring in the horizontal, vertical, or diagonal direction, with no intervening test location between them. For central clusters detected on 24-2C, the contribution of the 10 additional 24-2C test locations was further evaluated.

SD-OCT imaging was performed in all participants using the Nidek RS-330 (NIDEK Co., Ltd., Gamagori, Japan) according to a standardized protocol. Peripapillary RNFL was assessed using the Disc Map protocol (6.0 × 6.0 mm; 512 × 128), and average, superior, and inferior RNFL thicknesses (μm) were recorded for each eye. Macular imaging was performed using the Macula Map protocol (12.0 × 9.0 mm; 512 × 128). In the glaucoma module, superior and inferior ILM–IPL/INL thickness values (μm), representing mGCC thickness, were recorded for each eye, and the mean value was calculated as the arithmetic average of the superior and inferior measurements. Image quality was assessed using the device-derived signal strength index (SSI) together with segmentation integrity. Scans with an SSI ≥ 7/10 and without significant motion artifact, decentration, or segmentation error were included in the analysis; scans that did not meet these quality criteria were repeated during the same visit.

### Statistical Analysis

The sample size was determined by the number of consecutive eligible patients with OHT enrolled during the predefined recruitment period. To characterize the statistical sensitivity of this sample, a sensitivity power analysis was performed using G*Power (version 3.1.9.7) after enrollment was completed, based on the final sample size rather than on the observed effects. Given 60 paired eyes, a two-sided α of 0.05, and a power of 80%, the minimum detectable effect size was a standardized paired difference (dz) of approximately 0.38 for the Wilcoxon signed-rank test and a correlation coefficient of approximately |ρ| = 0.35.

The normality of continuous variables was assessed using the Shapiro–Wilk test. Because several variables were not normally distributed, nonparametric tests were used for paired comparisons; for comparability with previous studies, continuous variables are presented as mean ± standard deviation. Because of the paired study design, perimetric parameters obtained with the 24-2 and 24-2C protocols were compared within the same eye. Paired continuous perimetric variables, including MD, PSD, VFI, test duration, false-positive rate, the number of abnormal points at *p* < 0.05 and *p* < 0.01 within the central 10°, and central mean sensitivity, were compared between 24-2 and 24-2C using the Wilcoxon signed-rank test. The exact McNemar test was used for the paired comparison of normal/abnormal classification according to the central cluster criterion. The five-category GHT distributions were compared between 24-2 and 24-2C using the marginal homogeneity test. To assess structure–function relationships, associations between average peripapillary RNFL and mean mGCC thicknesses and central functional parameters were evaluated using Spearman correlation analysis. For the main comparisons, effect sizes are reported in the text. For continuous variables, these were the Hodges–Lehmann estimate of the median difference (24-2C minus 24-2) with its 95% confidence interval (CI) and r = Z/√N, where N is the number of pairs (0.1, small; 0.3, medium; ≥0.5, large). For the central cluster classification, these were the paired difference in proportions with its 95% CI (Newcombe method) and the discordant-pair odds ratio with its exact 95% CI. 95% CIs for single proportions were calculated using the Wilson score method. A *p* value < 0.05 was considered statistically significant.

## 3. Results

A total of 60 eyes were included in the study. The mean age of the participants was 50.4 ± 12.3 years, and women comprised the majority of the study population (66.7%). Mean intraocular pressure was 23.8 ± 2.6 mmHg. On structural assessment, mean RNFL and mGCC thicknesses were 105.6 ± 12.4 μm and 96.4 ± 6.7 μm, respectively. The demographic, clinical, and OCT characteristics of the participants are presented in Table 1.

No significant differences were observed between the 24-2 and 24-2C protocols in terms of the global visual field indices MD, PSD, and VFI (Table 2). Test duration was significantly longer with the 24-2C protocol than with the 24-2 protocol (167.4 ± 39.2 s vs. 141.6 ± 31.9 s; Hodges–Lehmann median difference 24.0 s, 95% CI 17.0–30.5; r = 0.70; *p* < 0.001). False-positive rates did not differ significantly between the two protocols (24-2, 4.9 ± 6.4% vs. 24-2C, 6.2 ± 5.7%; *p* = 0.168).

In the central visual field analysis, the number of abnormal test points at the *p* < 0.05 level within the central 10° was significantly higher with 24-2C than with 24-2 (3.81 ± 3.6 vs. 2.35 ± 2.4; median difference 1.0, 95% CI 0.5–2.0; r = 0.36; *p* = 0.006). The number of abnormal points at the *p* < 0.01 level within the central 10° was 0.75 ± 1.3 with 24-2 and 1.40 ± 2.9 with 24-2C; this difference did not reach statistical significance (*p* = 0.064). Central 10° mean sensitivity did not differ significantly between the two protocols (Table 2). The mean sensitivity of the 10 additional central test locations unique to the 24-2C protocol was 31.7 ± 1.8 dB.

The distribution of GHT results according to the two protocols is summarized in Table 3. Paired comparison of the five-category GHT classification showed no significant difference between the 24-2 and 24-2C protocols (marginal homogeneity test, *p* = 0.564).

According to the central cluster criterion, the 24-2 test was classified as abnormal in 9 of 60 eyes (15.0%; 95% CI 8.1–26.1), whereas an abnormal central cluster was identified in 19 eyes (31.7%; 95% CI 21.3–44.2) with 24-2C (Table 4). Among the 51 eyes classified as normal on 24-2, 14 (27.5%; 95% CI 17.1–40.9) showed a central cluster on 24-2C. Conversely, among the 41 eyes classified as normal on 24-2C, 4 (9.8%; 95% CI 3.9–22.5) showed a central cluster on 24-2. The proportion of eyes with an abnormal central cluster was significantly higher with 24-2C than with 24-2 (31.7% vs. 15.0%; paired difference 16.7 percentage points, 95% CI 2.8–30.0; discordant-pair OR 3.5, 95% CI 1.1–14.6; exact McNemar test, *p* = 0.031). A cluster that met the predefined criterion only after inclusion of the additional central test locations unique to 24-2C was identified in 6 eyes (10.0%).

In addition, the relationships between central functional findings and structural parameters were explored using peripapillary RNFL and mGCC thicknesses measured by SD-OCT. Neither average RNFL nor mean mGCC thickness showed a significant correlation with the number of abnormal points at *p* < 0.05 within the central 10° or with central 10° mean sensitivity for either the 24-2 or 24-2C protocol. Spearman ρ values were weak, ranging from −0.267 to 0.109 across all analyses, with no consistent directional pattern between the two protocols (all *p* > 0.05).

## 4. Discussion

In this study, no statistically significant differences were found between the 24-2 and 24-2C protocols in terms of global visual field indices (MD, PSD, and VFI). However, 24-2C identified a greater number of abnormal points at the *p* < 0.05 level within the central 10° and a higher rate of central cluster positivity than 24-2 (31.7% vs. 15.0%; *p* = 0.031). These findings suggest that 24-2C may provide additional information for the assessment of central visual function even in patients with OHT, a population conventionally considered to have no established perimetric loss. However, the clinical significance of this additional information should be interpreted cautiously, particularly in the absence of corresponding structural correlations with RNFL or mGCC measurements.

A major challenge in the management of OHT is that only a subset of patients will eventually progress to POAG, whereas unnecessary treatment carries its own burden in terms of cost, adverse effects, and adherence. Risk calculators based on risk factors identified in the Ocular Hypertension Treatment Study (OHTS) are widely used in clinical practice to individualize follow-up intervals and treatment decisions in patients with OHT [11,12]. In this context, current risk models rely predominantly on global perimetric indices, particularly PSD, and do not directly incorporate focal abnormalities within the central 10°. Therefore, how the additional central information provided by 24-2C might be integrated into future risk stratification models remains an important area for further investigation. In particular, prospective evaluation of whether the presence of a central cluster carries independent prognostic value in patients with OHT at moderate or high risk of progression may help clarify the potential contribution of 24-2C to clinical decision-making.

Previous studies have shown that macular damage in early glaucoma often follows an arcuate pattern and is related to the optic disc sector described as the “macular vulnerability zone.” However, such damage may be missed because of the relatively sparse sampling of the central 6° by the 24-2 test grid [5]. Similarly, several studies have reported central defects on 10-2 testing in glaucoma suspects and patients with early glaucoma despite a normal 24-2 result. In the study by Grillo et al., 24-2 testing was shown to miss a substantial proportion of OCT-confirmed macular damage [13]. Park et al. likewise reported that parafoveal scotomas were detected later during follow-up with 24-2 than with 10-2 testing, suggesting that reliance on 24-2 alone may delay recognition of progressive central damage [14]. An important finding of the present study was the significantly greater number of abnormal points at the *p* < 0.05 level within the central 10° with 24-2C (*p* = 0.006). However, the difference at the *p* < 0.01 level did not reach statistical significance. As previously noted, the 24-2C protocol includes 22 central test locations compared with 12 in the conventional 24-2 protocol. Therefore, the greater number of abnormal points detected with 24-2C may be partly attributable to the increased number of sampled locations, which inherently provides more opportunities to identify abnormalities. In addition, according to the predefined cluster criterion, the proportion of eyes with an abnormal central cluster was also significantly higher with 24-2C than with 24-2. To our knowledge, no previous study has directly compared 24-2 and 24-2C specifically in a pure OHT population. However, our findings appear more pronounced than those reported by Phu et al., who found that 24-2C detected more central clusters than 24-2 in a mixed cohort of patients with glaucoma and glaucoma suspects, although the difference was not statistically significant [10]. One possible explanation is that our study included a more homogeneous population consisting exclusively of patients with OHT. Differences in cluster definitions across studies may also contribute. Using a different cumulative criterion, Chakravarti et al. reported moderate-to-substantial agreement between 10-2 and 24-2C [9]. WuDunn et al. further noted that, because the 10 additional central locations in 24-2C were selected from points most frequently identified as abnormal on 10-2 testing, the 24-2C pattern may offer improved detection of central abnormalities compared with 24-2, particularly in early and preperimetric disease [3].

One of the distinctive findings of our study was that 24-2C identified central clusters in 27.5% of the 51 eyes that did not meet the central cluster criterion on 24-2, and in 6 of these eyes (10.0% of the entire cohort), cluster formation was directly attributable to the 10 additional central test locations unique to 24-2C. This finding suggests that some central functional abnormalities in eyes with OHT may be less adequately sampled by the standard 24-2 test pattern. Similarly, De Moraes et al. compared 24-2 and 10-2 testing in 108 eyes with OHT and found abnormal visual field clusters on 10-2 in 28 of 79 eyes (35.4%) that had been classified as normal on 24-2. In the same study, the proportion of abnormal tests in the overall OHT group increased from 26.8% with 24-2 to 39.8% with 10-2, which provides denser central sampling [6]. However, direct comparison of the detection rates between that study and ours is not appropriate because 10-2 samples 68 locations in the central field, whereas 24-2C adds only 10 central locations to the standard 24-2 pattern. Additionally, the contribution of additional central sampling may vary according to the study population. West et al., for example, reported limited evidence that adding 10-2 testing in patients with glaucoma and early 24-2 loss reveals additional previously undetected central defects, and suggested that 10-2 may be more appropriately reserved for follow-up in selected patients at higher risk of central progression [15]. Nevertheless, an important consideration in our study is that the central clusters identified were not confirmed by repeat visual field testing, leaving uncertainty as to whether they represent reproducible functional abnormalities or test-related variability. From a clinical perspective, whether the 14 eyes classified as normal on 24-2 but abnormal on 24-2C in our study represent a subgroup requiring closer surveillance can only be determined through longitudinal follow-up with serial functional testing and assessment of whether corresponding structural changes emerge over time.

No significant differences were observed between the two protocols in terms of the global indices MD, PSD, and VFI. This finding is consistent with previous studies comparing 10-2 with 24-2, as well as studies comparing 24-2C with both 24-2 and 10-2, and supports the general observation that global index performance is usually similar across different test grids [9,10,16]. However, it has also been reported that global index results may be influenced not only by the test grid but also by the thresholding strategy used [10]. With respect to test duration, the 24-2C protocol took approximately 26 s longer on average than 24-2 (167.4 vs. 141.6 s; *p* < 0.001). This difference is an expected consequence of the 10 additional central test locations included in 24-2C and is consistent with previous reports [17]. When the test algorithm is kept constant (SITA Faster), the difference in duration between 24-2C and 24-2 appears to be largely attributable to the 10 additional central points in the 24-2C pattern. When differences in testing algorithm are also introduced (SITA Standard vs. SITA Faster), the time difference becomes substantially greater. Indeed, in a study comparing SITA Standard 24-2 with SITA Faster 24-2C, the median difference between the two protocols was reported to reach 153 s [10]. The ability of SITA Faster to reduce test duration by approximately 53% compared with SITA Standard and by approximately 30% compared with SITA Fast is one of the main reasons why 24-2C can still be completed within a clinically acceptable time despite providing additional central sampling [18]. From this perspective, a potential clinical advantage of 24-2C is that it may provide additional information about the central field while reducing the extra time and workload that would be required if 10-2 were performed as a separate test. Direct comparisons between 10-2 and 24-2C have previously shown that 10-2 requires significantly longer testing time [8,19].

The lack of significant correlations between central functional parameters obtained with 24-2 and 24-2C and average RNFL or mGCC thicknesses in our study may be related to the imperfect correspondence between structural and functional changes, particularly at the early stages of disease. In glaucoma, structural and functional abnormalities do not necessarily emerge simultaneously; structural loss may precede detectable functional change in some eyes, whereas functional abnormalities may become apparent before measurable structural loss in others [20,21]. In eyes with OHT and preperimetric glaucoma, structure–function correlations between RNFL thickness and global visual field indices have been reported to be relatively weak, with stronger associations becoming evident as structural damage progresses [22]. In addition, our structure–function analysis was based on average RNFL and mGCC measurements rather than topographically matched structural and functional regions, which may also have limited our ability to detect significant correlations. Regionally matched structure–function analyses have been reported to be more useful for identifying localized associations [23]. Similarly, analyses of 24-2C have shown that central structure–function agreement may be better than with 24-2. However, when retinal ganglion cell displacement is taken into account, approximately half of the 24-2C test locations have been reported to fall outside the area covered by standard macular ganglion cell analysis [10]. The lack of a significant correlation in our study may be related to the early stage of disease and to methodological limitations arising from the use of global structural parameters to evaluate localized functional changes.

This study has several limitations. First, the single-center design and relatively limited sample size may restrict the generalizability of the findings. Moreover, no a priori sample size calculation was performed, and the sensitivity analysis indicated that the study was powered to detect only moderate or larger effects. Therefore, the non-significant findings, including those for PSD, VFI, and structure–function correlations, should not be interpreted as evidence of the absence of a difference or association. Second, the unequal number of central test locations between the two protocols represents an inherent methodological limitation when comparing the absolute number of abnormal points and central cluster detection rates. As the 24-2C protocol includes 10 additional central locations, the higher frequency of detected abnormalities may partly reflect the greater number of testing opportunities rather than a difference in diagnostic performance. Third, because of the cross-sectional design, the relationship between central clusters detected by 24-2C and subsequent conversion to POAG could not be evaluated. In addition, the lack of confirmation of these clusters with repeat 24-2C testing at a separate visit makes it difficult to determine whether the detected abnormalities represent true and reproducible functional changes or test variability. Another limitation is that, although test order was randomized and a rest interval was provided between examinations, a fatigue effect cannot be completely excluded because both tests were performed consecutively during the same visit. Previous studies have suggested that fatigue may adversely affect visual field performance during consecutive perimetric examinations [24]. However, studies involving repeated SITA Faster testing within the same visit have reported no systematic or clinically significant differences in visual field indices or pointwise sensitivity between successive examinations [25]. Nevertheless, potential fatigue and learning effects may have influenced the detection of central abnormalities in our study.

## 5. Conclusions

In conclusion, this study suggests that 24-2C may provide denser functional sampling of the central 10° in patients with OHT without altering global visual field indices and with an increase in testing time of approximately 26 s. However, in the absence of longitudinal validation and significant associations with OCT parameters, the clinical and prognostic significance of these abnormalities remains uncertain. Longitudinal studies are needed to determine the reproducibility of central abnormalities detected by 24-2C and their predictive value for conversion to POAG.

## Figures and Tables

**Table 1:** Demographic and baseline clinical characteristics.

Variable	All Participants (*n* = 60) ^1^
Age, years	50.4 ± 12.3
Sex, female/male, *n* (%)	40 (66.7)/20 (33.3)
Spherical equivalent, D	−0.09 ± 1.46
Best-corrected visual acuity, logMAR	0.03 ± 0.06
IOP, mmHg	23.8 ± 2.6
Central corneal thickness, μm	558.8 ± 33.5
C/D ratio	0.28 ± 0.08
Average RNFL thickness, μm	105.6 ± 12.4
Inferior RNFL thickness, μm	109.4 ± 15.3
Superior RNFL thickness, μm	101.8 ± 13.3
Mean mGCC thickness, μm	96.4 ± 6.7
Superior mGCC thickness, μm	94.1 ± 6.8
Inferior mGCC thickness, μm	98.8 ± 7.4

^1^ Data are presented as mean ± standard deviation, and categorical variables as *n* (%).

**Table 2:** Comparison of visual field parameters between the 24-2 and 24-2C protocols.

Parameter	24-2	24-2C	*p* *
MD, dB	−0.57 ± 1.62	−0.78 ± 1.37	0.130
PSD, dB	1.80 ± 0.61	2.02 ± 0.94	0.151
VFI, %	98.3 ± 1.5	97.9 ± 2.1	0.135
Test duration, s	141.6 ± 31.9	167.4 ± 39.2	**<0.001**
False-positive rate, %	4.9 ± 6.4	6.2 ± 5.7	0.168
Number of abnormal points at *p* < 0.05 within the central 10°	2.35 ± 2.4	3.81 ± 3.6	**0.006**
Number of abnormal points at *p* < 0.01 within the central 10°	0.75 ± 1.3	1.40 ± 2.9	0.064
Central 10° mean sensitivity, dB	31.8 ± 1.9	31.7 ± 2.4	0.128

* Wilcoxon signed-rank test. Data are presented as mean ± standard deviation. MD, mean deviation; PSD, pattern standard deviation; VFI, visual field index. Bold values indicate statistical significance (*p* < 0.05).

**Table 3:** Distribution of GHT results according to the 24-2 and 24-2C protocols.

GHT Result, *n* (%)	24-2	24-2C
Within normal limits	29 (48.3)	26 (43.3)
Borderline	13 (21.7)	16 (26.7)
Outside normal limits	17 (28.3)	16 (26.7)
Generalized reduction in sensitivity	1 (1.7)	1 (1.7)
Abnormally high sensitivity	0	1 (1.7)

The paired distribution across the five GHT categories was compared using the marginal homogeneity test; *p* = 0.564.

**Table 4:** Cross-tabulation of 24-2 and 24-2C visual field results according to the central cluster criterion.

Central Cluster Classification, *n* (%)		24-2C		
		Normal	Abnormal	Total
24-2	Normal	37 (61.7)	14 (23.3)	51 (85.0)
	Abnormal	4 (6.7)	5 (8.3)	9 (15.0)
	Total	41 (68.3)	19 (31.7)	60 (100)

Data are presented as *n* (%). The paired distributions were compared using the exact McNemar test; *p* = 0.031.

## Data Availability

The data presented in this study are available on request from the corresponding author. The data are not publicly available due to ethical restrictions.

## Abbreviations

The following abbreviations are used in this manuscript:

OHT	Ocular hypertension
POAG	Primary open-angle glaucoma
IOP	Intraocular pressure
RGC	Retinal ganglion cell
OCT	Optical coherence tomography
SD-OCT	Spectral-domain optical coherence tomography
RNFL	Retinal nerve fiber layer
mGCC	Macular ganglion cell complex
MD	Mean deviation
PSD	Pattern standard deviation
VFI	Visual field index
GHT	Glaucoma Hemifield Test
SITA	Swedish Interactive Thresholding Algorithm
ILM	Internal limiting membrane
IPL	Inner plexiform layer
INL	Inner nuclear layer
SSI	Signal strength index
OHTS	Ocular Hypertension Treatment Study
logMAR	Logarithm of the minimum angle of resolution
C/D	Cup-to-disc ratio
